# Data Science as a Core Competency in Undergraduate Medical Education in the Age of Artificial Intelligence in Health Care

**DOI:** 10.2196/46344

**Published:** 2023-07-11

**Authors:** Puneet Seth, Nancy Hueppchen, Steven D Miller, Frank Rudzicz, Jerry Ding, Kapil Parakh, Janet D Record

**Affiliations:** 1 Department of Family Medicine McMaster University Hamilton, ON Canada; 2 Department of Gynecology and Obstetrics Johns Hopkins University School of Medicine Baltimore, MD United States; 3 Division of Pediatric Gastroenterology, Hepatology, and Nutrition Department of Pediatrics Johns Hopkins University School of Medicine Baltimore, MD United States; 4 Faculty of Computer Science Dalhousie University Halifax, NS Canada; 5 Vector Institute for Artificial Intelligence Toronto, ON Canada; 6 Department of Computer Science University of Toronto Toronto, ON Canada; 7 Schulich School of Medicine and Dentistry Western University London, ON Canada; 8 Department of Medicine Georgetown University Washington, DC United States

**Keywords:** data science, medical education, machine learning, health data, artificial intelligence, AI, application, health care delivery, health care, develop, medical educators, physician, education, training, barriers, optimize, integration, competency

## Abstract

The increasingly sophisticated and rapidly evolving application of artificial intelligence in medicine is transforming how health care is delivered, highlighting a need for current and future physicians to develop basic competency in the data science that underlies this topic. Medical educators must consider how to incorporate central concepts in data science into their core curricula to train physicians of the future. Similar to how the advent of diagnostic imaging required the physician to understand, interpret, and explain the relevant results to patients, physicians of the future should be able to explain to patients the benefits and limitations of management plans guided by artificial intelligence. We outline major content domains and associated learning outcomes in data science applicable to medical student curricula, suggest ways to incorporate these themes into existing curricula, and note potential implementation barriers and solutions to optimize the integration of this content.

## The Emergence of Health Data Science and Artificial Intelligence

Health care is being swiftly transformed by the explosion of data sources and must rapidly transform data into information and actionable knowledge [1]. The sophistication of applications that use health data is increasing, ranging from simple medical calculators on smartphones, which calculate creatinine clearance [2], to clinical decision support (CDS) systems that use artificial intelligence (AI) to provide individualized lifetime risk information for certain cancers [3]. The introduction of large language models (LLMs) to the public sphere in 2022 significantly accelerated the discourse surrounding the potential integration of AI within health care and the risks and benefits involved [4]. The expanding volume and variety of health data and the increasing availability of algorithm- and AI-based tools also represents a trend in clinical decision-making that draws us nearer to the idea of actualizing data-driven personalized care. Hence, there is an urgent need to educate physicians, as informed curators and consumers of health data and related AI tools [5], regardless of specialty or location of practice [6].

Data science refers to an emerging interdisciplinary field that involves analyzing data through mathematical models, extracting knowledge, and deriving insights. Understanding the basic principles of data science as they pertain to health care delivery represents a foundation for the ability of the next generation of clinicians to safely and effectively work with sophisticated tools that use data. The Liaison Committee on Medical Education annually surveys medical schools regarding the inclusion of emerging topics. Based on the Association of American Medical Colleges’ Liaison Committee on Medical Education 2021-2022 Annual Medical School Questionnaire, 26% of medical schools surveyed included AI within either a required or elective course in that academic year, while for clinical informatics and precision medicine, these numbers were 79% and 66%, respectively [7]. These topics intersect with the applications of data science but do not independently provide a foundational layer of knowledge, nor are they consistently approached as a longitudinal theme in the education of students. While postgraduate programs and continuing education for advanced studies in data science for health care providers are available and while some medical schools are sporadically incorporating some related topics [8,9], the broad and rapidly evolving application of data science in health care demands a baseline competency in the subject for all clinicians.

We propose that a conceptual and practical framework of data science and its applications in health care should inform the drafting of a competency included in medical education. This viewpoint article outlines a framework and a list of topics that will facilitate medical students of today to become data-literate clinicians of tomorrow.

## Approach to Integration of Data Science Education Into Medical Curricula

### Overview

**Figure 1:**
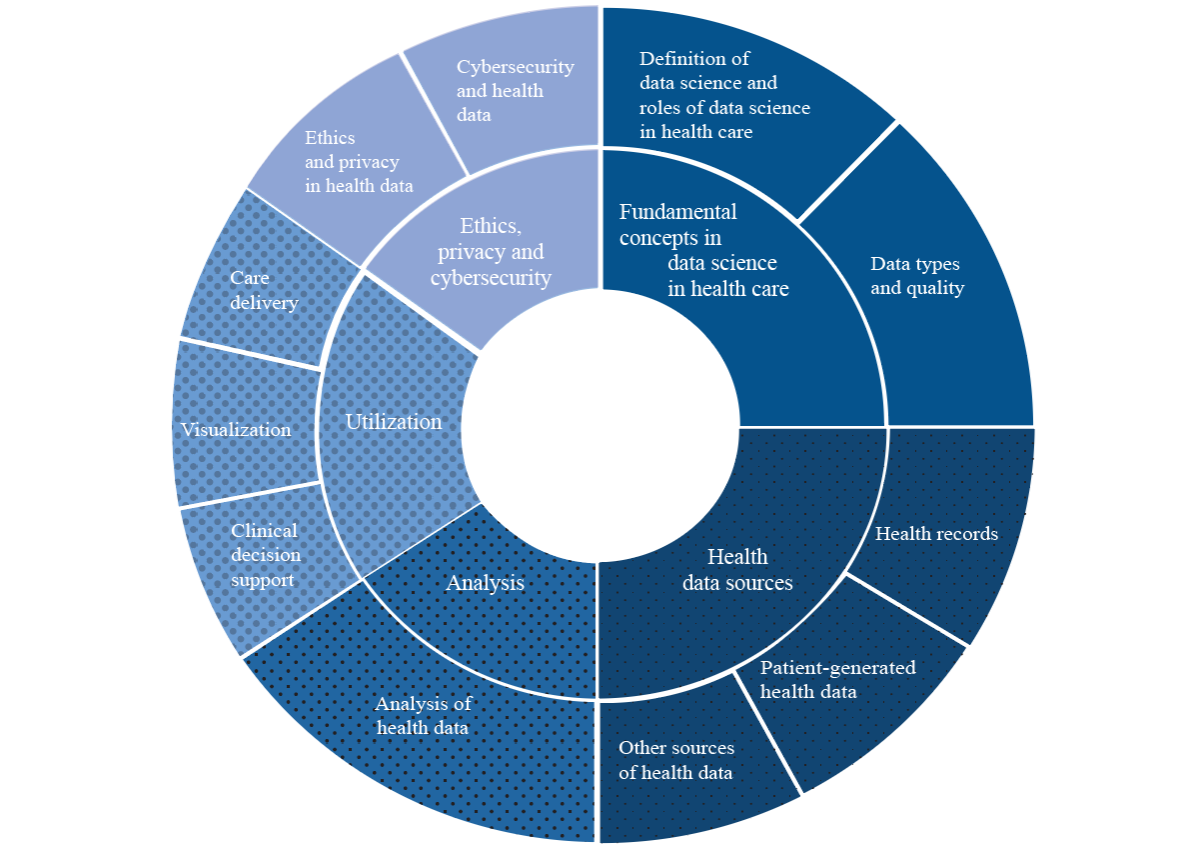
Visual representation of the proposed topics and subtopics for data science competency.

The topics outlined in detail in Table 1 and summarized in Figure 1 would provide a strong foundation for a stand-alone course on data science in health care for medical students, but integration, reinforcement, and application of the content throughout the curriculum will be essential to achieve meaningful learning outcomes. Here we outline suggestions and considerations for how each of the major topic areas in Table 1 may fit well into a focused anchor course positioned early in the Undergraduate Medical Education (UME) curriculum, paired with deliberate longitudinal integration across a medical education program. The content proposed has been derived through discussion among the subject matter experts in this paper along with a review of the Clinical Informatics Accreditation Council for Graduate Medical Education subspecialty curricula to extract topics relevant to health data science.

**Table 1:** A list of the proposed content domains and associated broad learning outcomes.

Topic, subtopic, and learning outcomes			Relevant AAMC^a^ competencies
**Fundamental concepts in data science in health care**			Interprofessional collaboration, knowledge for practice, personal and professional development, and systems-based practice
	**Definition of data science and roles of data science in health care**		
		Define data science as it applies to health care^b^ Describe the increasingly prominent and evolving role of data science in health care delivery and appreciate its relevance to clinical practice in any setting^b,c^	
	**Data types and quality**		
		Understand the various types of health data and considerations around data quality, focusing on the following: The idiosyncratic nature of health data and varied use of terminologies, such as Systemized Nomenclature of Medicine, International Classification of Diseases, and National Drug Code The role that free text plays as an important and contentious data type as compared to structured data The prevalence, causes, and implications of missing data Emerging data types, such as audio, video, genetic, transcriptomic and proteomic data, and images^b,d^ Describe the increasingly prominent and evolving role of data science in health care delivery and appreciate its relevance to clinical practice in any setting^b,c^	
**Health data sources**			Patient care, knowledge for practice, practice-based learning and development, systems-based practice, and interpersonal and communication skills
	**Health records**		
		Define and understand the utility of the various forms of health record systems in use today, such as electronic health records and personal health records^b,d^ Understand the role of health records in the generation, storage, and analysis of health data^b,c,d^ Compare and contrast the major types of databases and data schemas that are used in health records^b,c^ Local versus cloud storage Data warehouses versus data lakes	
	**Patient-generated health data**		
		Define patient-generated health data and understand its utility in health care delivery^b,d^ Explore the broad range of potential sources of patient-generated health data^c^	
	**Other sources of health data**		
		Understand other relevant sources of health data, and their benefits and limitations, including administrative data, billing and claims data, population health data, public health data, and “omics” data^b,d^ Explore how sources of health data may evolve over time^c^	
**Analysis**			Knowledge for practice, patient care, and practice-based learning and improvement
	**Analysis of health data**		
		Define and understand utility and rationale for use of traditional and novel methodologies of health data analysis, ranging from regression and nonregression methods to machine learning and neural networks^b,d^ Describe examples of novel methodologies of health data analysis using real-world data from various sources and explain potential applications^b,c^	
**Usage**			Knowledge for practice, practice-based learning and improvement
	**Visualization**		
		Understand the utility of health data visualization and the various ways in which health data can be presented^b,c^	
	**Care delivery**		
		Understand how artificial intelligence–based tools, such as large language models, can play a role in supporting both the administrative and clinical elements of health care delivery	
	**Clinical decision support**		
		Define what clinical decision support systems are, their reliance on data to generate accurate recommendations, and how they can be used in health care delivery^b,c^ Explain the role of the health care provider in evaluating the appropriateness of clinical decision support systems and assessing the factors that impact it, including the quality of the data set and inherent biases^d^	
**Ethics, privacy, and cybersecurity**			Professionalism, personal and professional development, and systems-based practice
	**Ethics and privacy in health data**		
		Describe privacy considerations involved in the collection, storage, and use of health data^d^ At a broad level, understand common mechanisms and potential consequences of breaches of privacy Understand the importance of ethical considerations in using health data^c,d^ Describe the importance of fairness toward equity, diversity, and inclusion in the design tools that use health data, such as machine learning algorithms Understand the role that learners and health care providers play in upholding ethical and privacy considerations^c,d^	
	**Cybersecurity and health data**		
		Define cybersecurity as it applies to health datad^d^ Develop an approach to maintaining competency in best practices in cybersecurity^c,d^	

^a^AAMC: Association of American Medical Colleges.

^b^AC: anchor course—a suggested approach or timing within medical curricula for a particular learning objective to be taught.

^c^CS: clerkship—a suggested approach or timing within medical curricula for a particular learning objective to be taught.

^d^PC: preclerkship—a suggested approach or timing within medical curricula for a particular learning objective to be taught.

### Fundamental Concepts in Data Science in Health Care

Early stages of a preclerkship curriculum represent an ideal opportunity to introduce fundamental concepts in data science, such as its definition, the important role it plays in the evolving landscape of health care delivery, and an overview of the types of health data and data quality. Delivery of this content can take place through a combination of an anchor course on the subject, along with brief required web-based modules created in collaboration with subject matter experts in health data science. These brief web-based modules may integrate well into preclerkship courses covering epidemiology, public health, and health system science.

Many medical schools incorporate longitudinal clinical experiences early in the preclerkship curriculum [10]. As students prepare to interact with clinical data in the electronic health record (EHR), reinforcement and further exploration of themes around data quality and data coding can be discussed. For example, a discussion on structured data (such as vital signs documented in discrete fields) versus unstructured data (such as free-text notes) may ideally take place when students are first being introduced to the use of EHRs in primary care, particularly in relation to their role in the management of chronic diseases such as type 2 diabetes. The integration of AI into health care and the increasing ability of LLMs to transform free text into structured data can be introduced. The importance of having structured data to observe trends necessary in making clinical decisions (such as looking at the trend of a patient’s blood sugar levels) or leveraging risk calculators (such as those for cardiovascular risk in patients with diabetes) should be highlighted.

### Health Data Sources

Learning about major types of databases and data schemas used in health records may fit best in an anchor course dedicated to clinical informatics or data science in health care. Such a focused course would allow students to explore key types of health records (such as EHRs and personal health records); the role they play in generating, storing, and analyzing health data; and the respective benefits and limitations of choosing local versus cloud storage of data.

Clerkship curricula can reinforce concepts in data sources deliberately through exploring case examples in small group discussions. For example, during a clinical rotation where students care for patients with rheumatologic conditions that require the tracking of a patient’s function and pain scores over time, the role of patient-reported outcomes through previsit questionnaires and data from consumer medical devices (such as step counters and sleep trackers) can be explored. As web-based care usage increases and medical device innovations evolve to provide novel types of patient-generated health data, medical trainees should be taught an approach to evaluate these novel data sources for appropriateness in integrating into clinical decision-making. Similarly, the expanding availability of “omics” data, which refers to comprehensive data sets generated from the analysis of different molecular aspects of biological systems such as genes, proteins, and metabolites, is important to discuss due both to its potential implications in advancing precision medicine and to its present practical and ethical limitations [11]. The abovementioned example of a patient with a rheumatologic condition could be a starting point for discussion around targeted biologic treatments based on genomic and proteomic screening, including AI-generated personalized predictive analytics.

Understanding the range of sources of health data is of particular importance in considering research questions and study design, with potential data sources including administrative data, billing and claims data, and population health data. The COVID-19 pandemic has resulted in the emergence of a number of such population health databases, where data are tracked on the administration of vaccines, viral testing, and contact-tracing, in many cases entirely outside of the patient health record [12]. The strengths and limitations of the various data sources are critical considerations, and teaching about data sources in the context of a research methods course represents an opportunity for integration of data science themes.

### Analysis of Health Data

Some traditional analytical methodologies, such as regression methods, may already be covered in existing components of the curriculum such as epidemiology or research methods [13,14]. An introduction to novel analytic tools and methodologies could be integrated into such existing courses or could be included in a data science anchor course.

Didactic sessions can explore how data science methodologies have evolved over time and what new capabilities are made possible through the use of advanced tools such as machine learning and neural networks, particularly in handling very large data sets to help produce insights tailored toward the needs of an individual patient (ie, precision medicine). It will be valuable for students to learn about the major machine learning models that exist (eg, unsupervised learning models, supervised learning models, and reinforcement learning models), along with their limitations, such as of a lack of explainability of results that is prevalent among such tools [15]. Students can consider specific case examples in an interactive, small group format, exploring benefits, barriers, and complexities that arise with implementing such rapidly evolving and sophisticated methods. An example for discussion would be the use of machine learning algorithms, such as general adversarial networks, to analyze and augment large data sets for the purposes of improving data quality and ensuring representation of an adequately broad spectrum of patient populations [16]. This improves the ability to build downstream applications that can better augment the ability of human radiologists through automated triaging, segmentation, and diagnosis of imaging modalities such as computed tomography and magnetic resonance imaging.

Students typically receive practical orientation to the particular EHR in use at core clinical clerkship sites [17], but these introductions could be made more robust with the exploration of data analytic tools embedded in the EHR, or tools being planned for near-term development. Clerkship directors can engage local subject matter experts in clinical informatics and data science to develop and implement interactive modules to learn about novel tools that are relevant locally. For example, a medical center may be adopting an application of machine learning in which optical character recognition automatically reads handwritten clinical notes. Students could learn about this new tool and consider its potential benefits (eg, facilitating medical documentation and coding; enhancing the quality of structured information in health records), as well as its inherent limitations.

Clinician educators may themselves lack knowledge on data science and the functioning of the tools available [18]. Learning outcomes around real-world usage may thus best be achieved through collaboration with data and IT professionals present at the clinical sites who could participate in small group sessions and simultaneously help raise awareness for clinician educators. In addition, subject matter experts in IT and data science can contribute to faculty session guide documents for small group work, outlining key teaching points that allow for faculty development of core clinical teachers who can then teach in these new content areas more independently.

### Usage

An initial introduction to the use of health data would educate students on the functional benefits and uses of health data as applied to clinical care delivery. This introduction to usage would fit well in an anchor course and should be delivered early enough in the preclerkship curriculum to allow for real-world examples to be highlighted within organ systems–based preclerkship courses. For example, when learning about renal function and discussing fluid balance, the crucial role of health data visualization within the electronic medical record (EMR) can be highlighted. Even in the preclerkship setting, educators can demonstrate how visual trending of relevant data parameters (including laboratory values such as serum creatinine and measurements such as blood pressure and weight) facilitates patient care.

Throughout the clinical rotations, as students encounter real-world examples of data visualization and CDS tools in the EMR, educators can ask students to notice and report on examples of important applications, benefits, and limitations of data use. Students should develop proficiency in using the EMR to visualize data that aids in clinical decision-making for their primary patients, starting with basics such as graphical trending of vital signs, laboratory test results, and medication dosing [19]. Students can explore existing CDS tools, recognizing important attributes of the data leveraged by these tools, including generalizability, data shift, and accuracy [20]. During an internal medicine rotation, students could discuss the implementation of an alert that uses machine learning algorithms to predict the risk of intensive care unit (ICU) transfer for an admitted inpatient on a medical ward [21] and the differential outcomes that may take place when data (such as the patient’s blood pressure or body temperature) are not collected accurately or recorded in a timely fashion. Similarly, a recent study revealed how the pandemic resulted in a data shift in the demographics of patients being admitted to the ICU, thereby reducing the accuracy of some sepsis prediction tools and leading to a surge in false positive alarm triggers [22]. Highlighting such differential impacts sets the stage for discussion around the validity of such algorithms in different patient populations and other biases that may impact accuracy of the CDS tool.

Similarly, the emerging role that novel AI tools will play in helping deliver both the administrative and clinical elements of health care is optimally discussed during clinical rotations and primarily through real-world examples, including attention to what may be on the horizon to alleviate present-day challenges; for example, growing evidence to support the ability of LLM-powered chatbots to serve as an interface for patient history taking and responding to common medical questions, providing both high-quality and empathetic responses [23]. It is appropriate to discuss this in the context of the strain on the health care system due to excessive amounts of administrative or nonclinical tasks assigned to health care providers [24].

### Ethics and Cybersecurity

Courses early in the preclerkship curriculum addressing medical ethics and professionalism present an opportunity for an introduction to concepts in privacy and ethics of health data usage. Prior to beginning any patient care activities, students complete the required Health Insurance Portability and Accountability Act (HIPAA) and patient privacy training, which address key components of privacy [25]. In later sessions focused on transition to clerkship, the curriculum should provide a more in-depth exploration of these topics, using specific case-based examples and interactive instructional methods. Finally, case studies during clinical rotations may be helpful in allowing students to apply this knowledge and integrate concepts into clinical practice. For example, students could consider a case study in which a pediatrician caring for a child with a mental health diagnosis discusses with parents the option of using data from the patient’s social media accounts to monitor mental health status, allowing for discussion of consent, privacy, access, bias, and authorization to use data from third-party platforms [26]. Additionally, the tools used by pediatricians to monitor mental health status themselves pose an opportunity to explore whether they are appropriate for the patient based on demographic factors, allowing for the exploration of fairness and equity [27].

As with topics in health data ethics, basic cybersecurity objectives should be included in modules required before students begin any patient care activity. More advanced cybersecurity topics may fit well in the transition to clerkship, with opportunities to reinforce and apply concepts as students transition between different clinical rotations.

## Framework for Teaching Data Science in the Context of the AI Revolution

**Figure 2:**
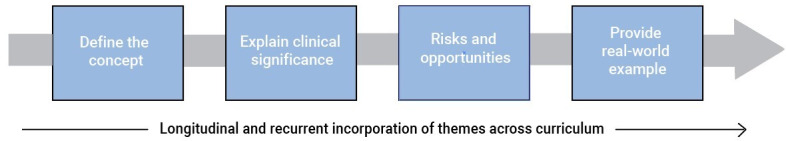
High-level framework for teaching data science concepts in undergraduate medical education.

Data science itself is a technical field of study that focuses greatly on statistics, database management, and computer programming. However, rather than focusing on the technical aspects of data science, we propose that each of the topics outlined above be approached using the framework outlined in Textbox 1 (with examples) and in Figure 2. The intent is to ensure that content remains clinically relevant and grounded in real-world examples.

Examples in the framework shown in Textbox 1 all refer to learning outcome 12, “Define and understand utility and rationale for use of traditional and novel methodologies of health data analysis, ranging from regression and non-regression methods, to machine learning and neural networks.”

**A high-level framework that can be applied to approaching the teaching of topics associated with data science to medical students.**

**Define the concepts introduced:**

Example: What is “health data analysis”?

**Explain its clinical significance:**

Example: What is the clinical utility of health data analytical methods? How and where are they used?

**Discuss risks and opportunities and how these may evolve:**

Example: What are the relevant risks and benefits associated with the use of various health data analytical methods? What might these look like in the next 5-10 years?

**Provide a real-world example of its utilization or an opportunity for students to engage in a hands-on assignment where applicable:**

Example: During the study of infectious diseases, discuss both traditional methods of calculating risk of sepsis vs novel algorithm-driven tools that can predict individualized risk for a patient.

## Considerations and Barriers

Effective implementation of these learning outcomes will require attention to the local context of the medical school, geography, and health system in which the students are being trained. For example, when discussing a topic such as privacy as it pertains to data, the content in US-based medical schools will be centered around HIPAA, the 21st Century Cures Act, and its applications, while those in Canadian schools will be appropriately focused on relevant provincial regulations, such as the Personal Health Information Protection Act of Ontario.

Given that data science and AI are in a highly active state of development and evolution, both the content within the topics and the topics themselves need to be continuously adapted. This is a notable departure from the typical approach to medical school curricula, which tend to retain a core focus on relatively stable content organized around traditional biomedical topics. Course directors and faculty are often subject matter experts in each particular, traditional field of medicine. These faculty members may not be familiar with certain emerging themes in medical education, including health data science and many others, such as structural competency, the history of race in medicine, sexual and gender diversity, and health effects of climate change. Hence, a critical component of the integration of health data science into medical education is an educational champion who oversees and updates the corpus of data science educational materials across the curriculum. Another essential factor in successful implementation is broader faculty development for the educators who will interact with students in classroom and clinical settings. To achieve focused faculty development efficiently for classroom-based sessions, small group case discussion guides can include key teaching points for core teaching faculty, allowing for just-in-time learning in content areas that may be new to some.

Another potential barrier is more ideological, namely the historical view of the clinical relationship as fiduciary [28] in which a physician acts in the best interest of a patient in an episode of care. The physician as the data scientist, responsible for using data to manage populations and selectively identify high-risk individuals for more intensified care based on computer algorithms, could be seen as a violation of the doctor-patient relationship. This is certainly an important concern, and data science instruction does need to be delivered within a curriculum that integrates humanism throughout. But arguing for humanism over the adoption of new technology and techniques ends up violating another sacred ethical principal of beneficence, namely that educating physicians on the use of data science at the bedside can make learners better, more effective clinicians. Just as medical educators might have taken a misguided resistance to integration of radiology into the curriculum with the concern that it would limit students’ learning of physical examinations, similar is the resistance to teaching the critical skill set of data analytics to the next generation of digitally savvy physicians.

## Conclusions

An understanding of the basic principles of data science and AI in modern health care must be considered a core competency for clinicians of today and the future. Medical training, along with education of other health care providers, must respond urgently to integrate these concepts into undergraduate medical curricula. Given the rapidly evolving nature of the field, data science education must be integrated into medical student curricula, iteratively reviewed to accommodate new developments, and accompanied by faculty development to support meaningful implementation to ensure that the next generation of physicians will be optimally prepared Data science–literate physicians will be able to wisely leverage the relative strengths of humans and machines, leading to the best outcomes for patients.

## Abbreviations

AI: artificial intelligence
CDS: clinical decision support
EHR: electronic health record
EMR: electronic medical record
HIPAA: Health Insurance Portability and Accountability Act
ICU: intensive care unit
LLM: large language model
UME: Undergraduate Medical Education
